# Fast genomic prediction of breeding values using parallel Markov chain Monte Carlo with convergence diagnosis

**DOI:** 10.1186/s12859-017-2003-3

**Published:** 2018-01-03

**Authors:** Peng Guo, Bo Zhu, Hong Niu, Zezhao Wang, Yonghu Liang, Yan Chen, Lupei Zhang, Hemin Ni, Yong Guo, El Hamidi A. Hay, Xue Gao, Huijiang Gao, Xiaolin Wu, Lingyang Xu, Junya Li

**Affiliations:** 1 Laboratory of Molecular Biology and Bovine Breeding, Institute of Animal Science, Chinese Academy of Agricultural Sciences, Yuanmingyuan West Road 2#, Haidian District, Beijing, 100193 China; 2 College of Computer and Information Engineering, Tianjin Agricultural University, Tianjin, China; 3 Animal Science and Technology College, Beijing University of Agriculture, Beijing, China; 4 Livestock and Range Research Laboratory, ARS, USDA, Miles City, MT USA; 5 Biostatistics and Bioinformatics, GeneSeek (A Neogen company), Lincoln, NE 68504 USA; 6 Department of Animal Sciences, University of Wisconsin, Madison, WI 53706 USA

**Keywords:** Bayesian models, Convergence diagnosis, Genomic prediction, High-performance computing, Tunable burn-in

## Abstract

**Background:**

Running multiple-chain Markov Chain Monte Carlo (MCMC) provides an efficient parallel computing method for complex Bayesian models, although the efficiency of the approach critically depends on the length of the non-parallelizable burn-in period, for which all simulated data are discarded. In practice, this burn-in period is set arbitrarily and often leads to the performance of far more iterations than required. In addition, the accuracy of genomic predictions does not improve after the MCMC reaches equilibrium.

**Results:**

Automatic tuning of the burn-in length for running multiple-chain MCMC was proposed in the context of genomic predictions using BayesA and BayesCπ models. The performance of parallel computing versus sequential computing and tunable burn-in MCMC versus fixed burn-in MCMC was assessed using simulation data sets as well by applying these methods to genomic predictions of a Chinese Simmental beef cattle population. The results showed that tunable burn-in parallel MCMC had greater speedups than fixed burn-in parallel MCMC, and both had greater speedups relative to sequential (single-chain) MCMC. Nevertheless, genomic estimated breeding values (GEBVs) and genomic prediction accuracies were highly comparable between the various computing approaches. When applied to the genomic predictions of four quantitative traits in a Chinese Simmental population of 1217 beef cattle genotyped by an Illumina Bovine 770 K SNP BeadChip, tunable burn-in multiple-chain BayesCπ (TBM-BayesCπ) outperformed tunable burn-in multiple-chain BayesCπ (TBM-BayesA) and Genomic Best Linear Unbiased Prediction (GBLUP) in terms of the prediction accuracy, although the differences were not necessarily caused by computational factors and could have been intrinsic to the statistical models per se.

**Conclusions:**

Automatically tunable burn-in multiple-chain MCMC provides an accurate and cost-effective tool for high-performance computing of Bayesian genomic prediction models, and this algorithm is generally applicable to high-performance computing of any complex Bayesian statistical model.

**Electronic supplementary material:**

The online version of this article (doi:10.1186/s12859-017-2003-3) contains supplementary material, which is available to authorized users.

## Background

Genomic predictions have been proposed as a method of providing accurate estimates of the genetic merits of breeding animals using genome-wide SNP markers [1]. This new technology does not require the actual phenotyping of breeding candidates and therefore offers great promise for traits that are difficult or expensive to measure, such as carcass traits [2]. A noted feature of genomic predictions is that selection can be performed on breeding candidates at birth or young ages, which in turn accelerates genetic improvement progress more rapidly than conventional breeding approaches in farm animals [3–6].

Many genomic prediction models have been proposed, such as the Genomic Best Linear Unbiased Prediction (GBLUP) [7] and Bayesian alphabets [1, 8]. Bayesian genomic models are widely used [8–11], although complex calculations are required for Bayesian models implemented via Markov chain Monte Carlo (MCMC) and may take hours, days or even weeks to complete. Hence, parallel computing for genomic predictions is of importance for applying genomic selection in practice [12]. However, parallelization in MCMC is difficult because the procedure is iterative in the sense that simulating the next value of the chain depends on the current value, which violates Bernstein’s condition of independence for parallel computing [13]. This problem increases the difficulty of delivering parallelism for a single Markov chain. Thus, Wu et al. proposed the use of a multiple-chain MCMC method to calculate Bayesian genomic prediction models [14].

Running multiple-chain MCMC provides a naïve yet efficient form of parallel computing for Bayesian genomic prediction models, although the speedup in computing is limited by the burn-in requirement, which is included to give the Markov chain time to reach equilibrium distribution. A burn-in period corresponds to the first *n* samples during the MCMC, and these samples are discarded after the burn-in is initiated from a poor starting point to the period before each chain moves into a high probability state. Often, the length of the burn-in is assumed to be one-tenth or one-fifth of the entire length of the MCMC iterations or even half of the total iterations [1, 8–10]. This rule of thumb is often used for the sake of convenience but is not necessarily optimal for computing.

In the present paper, a multiple-chain MCMC computing strategy utilizing automatic tuning of the burn-in period was proposed and demonstrated with two Bayesian genomic prediction models (BayesA and BayesCπ). Using this strategy, a convergence diagnosis based on Gelman and Rubin [15] was conducted periodically in accordance with the multiple-chain situation. The burn-in period ended as soon as the convergence criteria were met, and posterior samples of unknown model parameters were then collected to perform statistical inferences. This strategy was assessed on a simulation data set and applied to genomic predictions of four quantitative traits in a Chinese Simmental population.

## Methods

### Simulation data

The simulated data set consisted of 1000 animals in scenario 1 and scenario 2 and 2000 animals in scenario 3, with each presenting a phenotype and genotypes on five chromosomes. The GPOPSIM software package [16] was used to generate the simulation data set, including the markers and QTLs based on a mutation-drift equilibrium model in the three scenarios. In scenario 1, the heritability of the trait was set at 0.1, and each chromosome had 4000 markers. In scenario 2, the heritability was 0.5, and each chromosome had 10,000 markers. In scenario 3, the heritability was 0.3, and each chromosome had 40,000 markers. In each of the three scenarios, 200 QTLs were simulated. The mutation rate of the markers and QTLs was set at 1.25 × 10^−3^ for each generation.

### Real phenotype and genotype data

The experimental population consisted of 1302 Simmental cattle born between 2008 and 2013 in Ulgai, Xilingol League, Inner Mongolia, China. After weaning, all cattle were transferred to the Beijing Jinweifuren farm and raised under the same nutritional and management conditions. Each animal was evaluated regularly for growth and development traits until slaughter at between 16 and 18 months of age. At slaughter, the carcass traits and meat quality traits were assessed according to the Institutional Meat Purchase Specifications [17] for Fresh Beef Guidelines. The quantitative traits used in the present study included carcass back fat thickness (CBFT), strip loin weight (SLW), carcass weight (CW), and average daily gain (ADG). Prior to the genomic predictions, the phenotypes were adjusted for systematic environmental factors, which included the farms, seasons and years, and age at slaughter, using a linear regression model. The genetic and residual variances for each of the four traits were estimated by restricted maximum likelihood (REML) based on equivalent animal models.

Each animal was genotyped by an Illumina Bovine 770 K SNP BeadChip. SNP quality control was conducted using PLINK v1.07 software [18], which excluded SNPs under the following categories: 1) SNPs on the X and Y chromosomes, 2) SNPs with minor allele frequencies less than 0.05, 3) SNPs with > 5% missing genotypes, and 4) SNPs that violated Hardy-Weinberg equilibrium (*p* < 10^−6^). After data cleaning, 1217 Simmental cattle remained for subsequent data analyses, and each had genotypes on up to 671,220 SNPs on 29 autosomes.

### Statistical model

Adjusted phenotypes were described by the following linear regression model:

1 $$ {y}_i=\upmu +{\sum}_{j=1}^M{X}_{ij}{\alpha}_j+{e}_i $$

where *y*_*i*_ is an adjusted phenotype for individual *i*, *M* is the number of SNPs, *μ* is the overall mean of the traits, *a*_*j*_ is the additive (association) effect of the *j-*th SNP, *X*_*ij*_ is the genotype (0, 1, or 2) of the *j*-th SNP observed on the *i*-th individual, and *e*_*i*_ is the residual term.

#### BayesA

The BayesA model [1] assumed a priori a normal distribution for SNP effects, with zero mean and SNP-specific variances denoted by ${\upsigma}_j^2$, where *j* = 1, 2, …, *M*. The variances of SNP effects were independent of one another, and each followed an identical and independently distributed (IID) scaled inverse chi-square prior distribution, $\mathrm{p}\left({\upsigma}_j^2\right)={\upchi}^{-2}\Big({\upsigma}_j^2\mid \nu, {S}^2$), where ν is the degree of freedom parameter and *S*^2^ is the scale parameter, both of which are assumed to be known. Thus, the marginal prior distribution of each marker effect, $\mathrm{p}\left({\upalpha}_j|\nu, {S}^2\right)=\int N\left({\upalpha}_j|0,{\upsigma}_j^2\right){\upchi}^{-2}\left({\upsigma}_j^2|\nu, {S}^2\right)d{\upsigma}_j^2$, was a *t*-distribution [19].

#### BayesCπ

The BayesCπ model [8] assumed a priori that each SNP effect was null with probability π or followed a normal distribution, $N\left(0,{\sigma}_a^2\right)$, with probability 1-π.

2 $$ \left.{a}_j\right|\pi, {\sigma}_a^2\sim \left\{\begin{array}{c}N\left(0,{\sigma}_a^2\right)\kern0.5em with\kern0.17em probability\left(1-\pi \right)\\ {}0\kern0.5em with\kern0.17em probability\;\pi \end{array}\right. $$

In the above, ${\sigma}_a^2$ is a variance common to all non-zero SNP effects, and it is assigned a scaled inverse chi-square prior distribution, ${\chi}^{-2}\left({v}_a,{s}_a^2\right)$. The value of *π* in the model is unknown, and it is inferred based on the prior distribution of *π*, which is considered uniform between 0 and 1, or *π*~Uniform(0, 1).

#### GBLUP

GBLUP [7] can be considered a re-parameterization of the Bayesian RKHS (reproducing kernel Hilbert spaces) regression [20]. In RKHS, each SNP effect is assumed to follow a normal distribution with a zero mean and common variance; and in GBLUP, genomic estimated breeding values (GEBVs) are assumed to follow a normal distribution $\boldsymbol{u}\sim \mathrm{N}\left(0,\mathbf{G}{\upsigma}_u^2\right)$, where **G** is a *n* × *n* genomic (co)variance matrix that is formulated as follows [7]:

3 $$ G=\frac{{\boldsymbol{XX}}^{\hbox{'}}}{2{\sum}_{i=1}^n{q}_i\left(1-{q}_i\right)}, $$

where *n* is the number of SNPs, *q*_*i*_ is the frequency of an allele of SNP *i*, and **X** is a centered incidence matrix of SNP effects, which are corrected for allele frequencies. The additive genetic variances and residual variances of the four traits were estimated by REML based on an animal model equivalent to (1).

### Tunable versus fixed burn-in multiple-chain MCMC

#### Tunable burn-in multiple-chain MCMC

In multiple-chain MCMC simulations, the following processes occur. Assume that we want to estimate some target distribution *p*(*X*) but cannot directly draw samples of X from *p*(*X*). Instead, a Markov chain *X*_0_, *X*_1_, … can be generated that converges to *p*(*X*) at equilibrium via a transition density *u*(*X*_*t* + 1_| *X*_*t*_). Now, let there be *i* = 1, 2, …, *K* parallel chains, with each initialized and burned-in independently for *B*_*i*_ updating steps before more samples are drawn at intervals. As *K* → ∞ and all *B*_*i*_ → ∞, the ensemble is ergodic (i.e., tending in the limit) to *p*(*X*) [14].

To assess the convergence of multiple parallel chains simulated for each model, both the inter-chain and within-chain variances were calculated for each selected model parameter, e.g., *x*. Briefly, the inter-chain variance *I* was calculated as follows:

4 $$ I=\frac{n}{m-1}{\sum}_{i=1}^m{\left({\overline{x}}_i-\overline{x}\right)}^2 $$

The within-chain variance W was determined as follows:

5 $$ W=\frac{1}{m}{\sum}_{i=1}^m{s}_i^2. $$

where ${s}_i^2=\frac{1}{n-1}{\sum}_{j=1}^n{\left({x}_{ij}-{\overline{x}}_i\right)}^2$, ${\overline{x}}_i=\frac{1}{n}{\sum}_{j=1}^n{x}_{ij}$, and $\overline{x}=\frac{1}{m}{\sum}_{i=1}^m{\overline{x}}_i$. Then, the marginal posterior variance of *x* was estimated by a weighted average of *W* and *I* as follows:

6 $$ \widehat{\mathit{\operatorname{var}}}(x)=\frac{n-1}{n}W+\frac{1}{n}I. $$

Under the assumption that the starting distribution of *x* was appropriately over-dispersed, the above quantity tended to overestimate the marginal posterior variance but was unbiased under stationarity (i.e., when the starting distribution equals the target distribution) or within the limit, *n* → ∞.

Following Gelman et al. [21], we assessed convergence by estimating the factor by which the scale of the current distribution for *x* might be reduced if the posterior simulation were continued within the limit, *n* → ∞. This potential scale reduction was estimated by the following shrink factor:

7 $$ r=\sqrt{\frac{\widehat{\mathit{\operatorname{var}}}(x)}{W}} $$

which reduced to 1 as *n* → ∞. A high-scale reduction indicated that proceeding with more simulations could further improve the inference about the target distribution of the model parameter. To run multiple-chain MCMC simulations, a collection of shrink factors was obtained, R = (*r*_0_, *r*_1_, …, *r*_*N −* 1_), where *N* is the length of a chain, R^(j)^_=(_*r*_(j *−* 1) × p_, *r*_(j *−* 1) × p + 1_, …, *r*_j × p *−* 1)_ is the *j*th subsection of R, and *p* is the length of R^(j)^. Let *T* be a threshold that was arbitrarily provided; the mean ${\overline{\mathrm{r}}}^{(j)}$ and standard deviation *S*^(*j*)^ were calculated as follows:

8 $$ {\overline{r}}^{(j)}=\frac{1}{p}{\sum}_{i=0}^{p-1}{r}_{\left(j-1\right)\times p+i}, $$

9 $$ {S}^{(j)}=\sqrt{\frac{1}{p}{\sum}_{i=0}^{p-1}{\left({r}_{\left(j-1\right)\times p+i}-{\overline{r}}^{(j)}\right)}^2}. $$

The multiple chains were considered to converge in the *j*th subsection when $\left|{\overline{r}}^{(j)}-1\right|<T$ and *S*^(*j*)^ < *T*.

In the simulation study, each of the parallel MCMC chains was initiated independently. Then, the convergence diagnosis during burn-in used samples from each parallel chain to determine the convergence state of these chains. The end of burn-in iterations occurred when the convergence criteria were met. Then, the simulated posterior samples were collected to calculate the posterior summary statistics of the model parameters of interest, which were subject to the thinning of the MCMC chains.

For the Simmental cattle data set, we evaluated genomic prediction accuracies (GPAs) by running up to 16 parallel chains for both TBM-BayesA and TBM-BayesCπ, and the results were compared with those obtained from GBLUP.

#### Speedup ratio

According to Amdahl’s law [22], the speedup ratio of multiple-chain MCMC over that of single-chain MCMC was calculated as follows:

10 $$ \mathrm{S}\left(\mathrm{K}\right)=\frac{{\mathrm{N}}_{\mathrm{T}}}{{\mathrm{N}}_{\mathrm{burn}-\mathrm{in}}+\left({\mathrm{N}}_{\mathrm{T}}-{\mathrm{N}}_{\mathrm{burn}-\mathrm{in}}\right)/\mathrm{K}} $$

where N_burn-in_ is the number of burn-in iterations that cannot be parallelized, N_T_ is the total number of MCMC iterations, and *K* is the number of computer cores available for running multiple-chain Markov chains in parallel. The parallel computing efficiency was assessed as follows:

11 $$ E=S(K)/K $$

Evidently, when E = 1, the parallel computing scales linearly with the number of cores used for computing; thus, *S(K)* = K. However, because of the non-parallelizable burn-ins, the parallel computing efficiency is upper bounded by *N*_*T*_/*N*_*burn* − *in*_ (as *K* → ∞).

#### Parameter setting

Fixed burn-in multiple-chain MCMC jobs were also run on the simulation data, with the length of burn-in set at one-tenth of the total sequential MCMC iterations in scenario 2 and at one-fifth of the total sequential MCMC iterations in scenarios 1 and 3. To assess the effect of the burn-in length, we ran 50,000 iterations for each chain of fixed burn-in multiple-chain BayesA (FBM-BayesA), which included burn-ins of 2000, 4000, 6000, 8000, and 10,000 iterations in scenario 2. Threshold *T* was set to 0.001 in this study.

### Evaluation of genomic prediction accuracy

The GEBVs were calculated as the sum of all SNP effects of each individual (say *i*) as follows:

12 $$ {GEBV}_i={\sum}_j{X}_{ij}{g}_j $$

where *X*_*ij*_ is a genotype (coded 0, 1, or 2) for SNP *j* of animal *i* and *g*_*j*_ is the estimated genetic effect of the *j*th SNP.

The GPA relative to that of phenotypic selection was calculated as $r/\sqrt{h^2}$, where *r* is Pearson’s correlation between GEBVs and true breeding values in the simulation study or Pearson’s correlation between GEBVs and adjusted phenotypes. This criterion of relative genomic prediction accuracy (RGPA) was used so that the GPAs were comparable regardless of their respective heritabilities [23].

A fivefold cross-validation [24] was used to evaluate the genomic predictions in the Simmental data set. Briefly, the entire data set of 1217 Simmental cattle was randomly divided into five approximately equal subsets. Then, four subsets were used to estimate the SNP effects (i.e., training), and the remaining subset was used for testing the GPA (i.e., validation). The above process was rotated five times until each subset was used for testing once and only once. For each trait, fivefold cross-validations were randomly duplicated 10 times, and the GPA for each trait was calculated as the average of GPAs across the ten replicates.

### Computer system

The calculations were conducted on an HP ProLiant DL585 G7 (708686-AA1) server, which was equipped with an AMD Opteron 6344 (2.6G Hz) CPU, 272 G of memory and an L2 cache size of 4 M and an L3 cache size of 16 M. The operating system was Microsoft Windows. A C program with Message Passing Interface (MPI) for parallel computing was developed to implement the aforementioned multiple-chain MCMC. MPICH2 is an open source MPI implementation and a standard for message passing in parallel computing, and it is available freely (http://www.mpich.org/downloads). The Integrated Development Environment that we used is Dev-C++ 5.1, which is available freely at the following link: http://www.bloodshed.net/index.html.

## Results

### Simulation studies

#### Speedup ratios

**Fig. 1:**
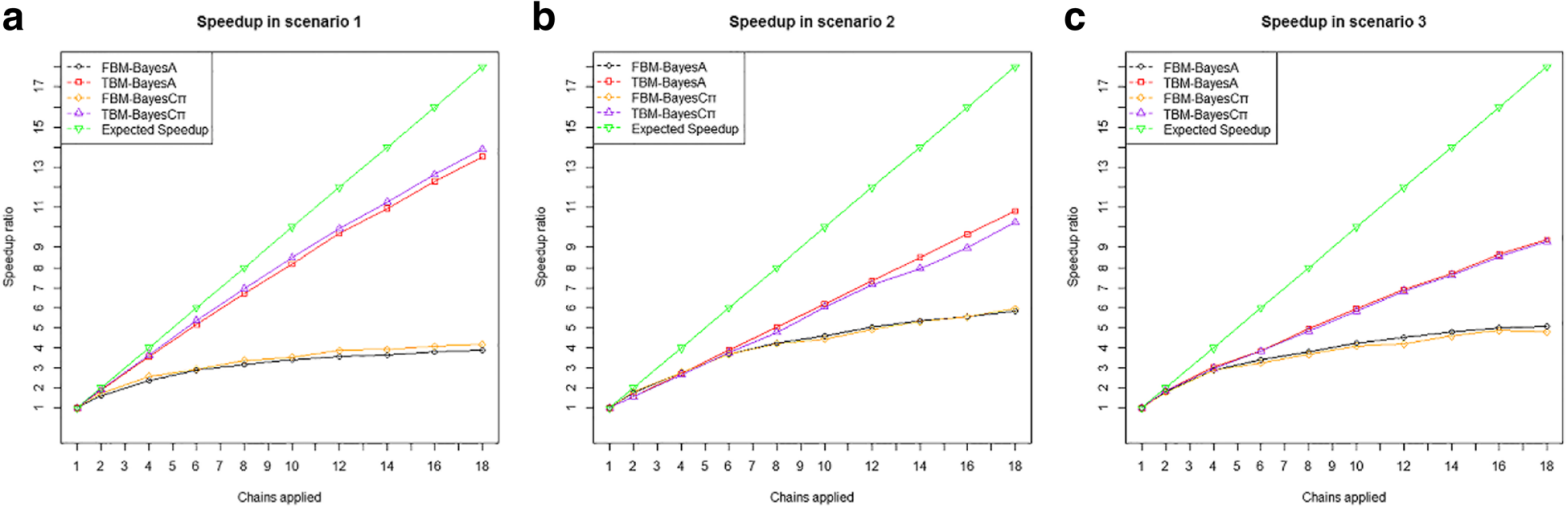
Speedup ratios of parallel MCMC (>1 chain) over sequential MCMC (1 chain) in simulation studies under the three scenarios: **a** Scenario 1 (*h*^2^ = 0.1; 4000 SNPs; 200 QTLs), **b** Scenario 2 (*h*^2^ = 0.1; 10,000 SNPs; 200 QTLs), and **c** Scenario 3 (*h*^2^ = 0.1; 40,000 SNPs; 200 QTLs). Expected speedup ratios were calculated under the assumption that MCMC chains were 100% parallelizable (i.e., without burn-in)

Running multiple chains of genomic prediction models led to substantially reduced computing time compared with running a single chain (Additional file 1: Tables S1–S3 and Figures S9–S11). The speedups increased non-linearly with the number of parallelized chains or available computer cores (Fig. 1) because of the non-parallel burn-ins, and perfect speedups were not practically observed when calculating these Bayesian genomic prediction models. In scenario 1, for example, the speedup obtained by TBM-BayesA was 1.86 when running two parallel chains and was 13.63 when running 18 parallel chains. However, the speedup obtained by FBM-BayesA increased from 1.57 when running two chains to 3.79 when running 18 parallel chains. For the results obtained with 18 parallel chains, the speedups were approximately between 3 and 6 when running fixed burn-in MCMC and between 10 and 14 when running tunable burn-in MCMC. More precisely, TBM-BayesA had considerably greater speedups than those of FBM-BayesA, and the speedups by TBM-BayesA scaled better than those by FBM-BayesA. Similar trends were found in the comparison of computing time between TBM-BayesCπ and FBM-BayesCπ (fixed burn-in, multiple-chain BayesCπ). Thus, tunable burn-in MCMC had greater parallel computing efficiencies than fixed burn-in MCMC because the use of automatic convergence diagnosis and tuning of burn-ins effectively shortened the computing time by TBM-BayesA (or TBM-BayesCπ), resulting in increased speedups in computing time with tunable burn-ins. We also noted that the loss of parallel computing efficiency relative to an assumedly perfect speedup increased with the model dimension, which is proportional to the number of SNPs in the genomic prediction models (Fig. 1).

Theoretically, the speedups achieved by running multiple-chain MCMC are limited by the length of non-parallel parts (i.e., burn-ins). Frequently, the rule of thumb for the length of burn-in tends to result in far more burn-in iterations than are required. Thus, with automatic tuning of the convergence diagnosis on multiple-chain MCMC, the burn-in length can be drastically reduced, resulting in greater speedups in computing. With all other factors equal, the speedup obviously increased with a greater number of chains (or CPU cores) running in parallel (Fig. 1).

##### Estimated model (SNP) effects

**Fig. 2:**
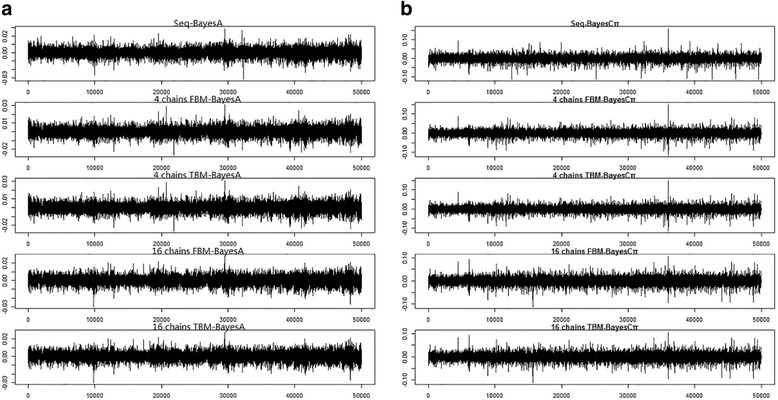
Trace plots of the simulated residual variance using various computational forms of (**a**) BayesA and (**b**) BayesCπ. The total length of MCMC was 50,000 iterations, and the length of the fixed burn-in period was 5000 iterations

Various computing forms of the same genomic prediction models essentially generated highly comparable estimated model effect results. Trace plots of the residual variance obtained by various computational approaches are shown in Fig. 2. Each chain mixed very well, and all were centered near zero.

**Fig. 3:**
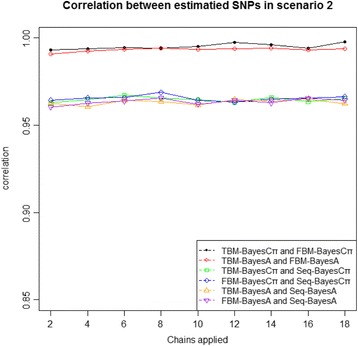
Correlations of the estimated SNP effects in simulation scenario 2. Seq-BayesA = sequential BayesA; Seq-BayesCπ = sequential BayesCπ; FBM-BayesA = fixed burn-in, multiple-chain BayesA; TBM-BayesA = tunable burn-in, multiple-chain BayesA; FBM-BayesCπ = fixed burn-in, multiple-chain BayesCπ; TBM-BayesCπ = tunable burn-in, multiple-chain BayesCπ

The estimated SNP effects were also highly comparable among various forms of calculating the same genomic prediction models. In parallel computing, between 2 and 18 chains (with an increment of two chains) were run for each model, and the posterior mean of a SNP effect was calculated as the average of all saved posterior samples from all the chains running for that model. In sequential computing, a SNP effect was calculated as the average of all the saved posterior samples from the single chain. The results showed that the correlations of estimated SNP effects, such as in scenario 2, were greater than 0.80 between parallel MCMC and sequential MCMC and even greater than 0.90 between tunable burn-in MCMC and fixed burn-in MCMC (Fig. 3). For example, the correlations of estimated SNP effects were from 0.901 to 0.908 between FBM-BayesA and TBM-BayesA. Similar results were obtained in scenarios 1 and 3 (data not presented). Thus, the observed differences in estimated SNP effects for the same method were attributable to Monte Carlo errors, and they were essentially made trivial as soon as the MCMC chains converged to the expected stationary distributions.

##### GEBVs and GPAs

The GEBV of each animal was calculated as the sum of all SNP effects for that animal. The results showed that the GEBVs obtained from various forms of calculating the same genomic prediction models were almost identical and presented correlations that were greater than or close to unity (> 0.99). The GEBVs obtained from different models were also highly correlated but with some noticeable differences. These differences did not result from the use of varied computational strategies but reflected the use of different statistical models (and the underlying model assumptions).

Similarly, the GPAs were also analogous between different computational forms of the same model, regardless of the number of MCMC chains and types of burn-in mechanisms, although noticeable differences were observed in the GPAs between different statistical models (Tables 1, 2 and 3). In simulation scenario 1, for example, the GPA was approximately 0.523 for the various forms of BayesA with either fixed or automatically tunable burn-in periods running between 1 and 18 chains. However, the GPA obtained by various computing forms of BayesCπ varied only slightly between 0.630 and 0.632 (Table 1). Similar trends were observed in simulation scenarios 2 and 3. As a comparison, GPAs were also obtained using GBLUP; however, these values were mostly lower than the GPAs obtained from the various computing forms of BayesA and BayesCπ, although BayesA models in scenario 1 were exceptions (Table 1). Again, the slight differences in GPA among the various computing forms of the same genomic prediction models were caused by Monte Carlo errors in the simulation of posterior samples of SNP effects, whereas the differences in GPA between the various statistical models were not necessarily computational but were attributable to intrinsic differences between the methods per se.

**Table 1:** Genomic predictive accuracies obtained using FBM-BayesA, TBM-BayesA, FBM-BayesCπ, TBM-BayesCπ, and GBLUP in Scenario 1

Chains	FBM-BayesA	TBM-BayesA	FBM-BayesCπ	TBM-BayesCπ	GBLUP
1	0.5239	0.5231	0.6316	0.6317	0.6016
2	0.5227	0.5229	0.6301	0.6296	
4	0.5230	0.5229	0.6304	0.6314	
6	0.5230	0.5230	0.6310	0.6304	
8	0.5231	0.5232	0.6313	0.6311	
10	0.5232	0.5231	0.6309	0.6307	
12	0.5228	0.5232	0.6315	0.6305	
14	0.5230	0.5231	0.6306	0.6313	
16	0.5230	0.5231	0.6307	0.6303	
18	0.5230	0.5231	0.6310	0.6311	

*FBM-BayesA* fixed burn-in, multiple-chain BayesA, *TBM-BayesA* tunable burn-in, multiple-chain BayesA, *FBM-BayesCπ* fixed burn-in, multiple-chain BayesCπ, *TBM-BayesCπ* tunable burn-in, multiple-chain BayesCπ, and *Chains* number of parallel MCMC running for each genomic prediction model. Simulation parameters are as follows: population size = 1000; number of QTL = 200; heritability = 0.1; number of chromosomes = 5; and number of markers per chromosome = 4000

**Table 2:** Genomic predictive accuracies obtained using FBM-BayesA, TBM-BayesA, FBM-BayesCπ, TBM-BayesCπ, and GBLUP in Scenario 2

Chains	FBM-BayesA	TBM-BayesA	FBM-BayesCπ	TBM-BayesCπ	GBLUP
1	0.846777	0.846761	0.937837	0.937312	0.833173
2	0.846731	0.846709	0.937741	0.937161	
4	0.846797	0.846855	0.937449	0.937160	
6	0.846904	0.846855	0.938563	0.938017	
8	0.846559	0.846884	0.938305	0.938319	
10	0.846770	0.846812	0.938232	0.938763	
12	0.846862	0.846824	0.938997	0.938347	
14	0.846814	0.846832	0.938535	0.938426	
16	0.846844	0.846657	0.938852	0.938385	
18	0.846820	0.846854	0.938823	0.938210	

Simulation parameters are as follows: population size = 1000; number of QTL = 200; heritability = 0.5; number of chromosomes = 10; and number of markers per chromosome = 5000

**Table 3:** Genomic predictive accuracies obtained using FBM-BayesA, TBM-BayesA, FBM-BayesCπ, TBM-BayesCπ, and GBLUP in Scenario 3

Chains	FBM-BayesA	TBM-BayesA	FBM-BayesCπ	TBM-BayesCπ	GBLUP
1	0.7717	0.7717	0.8415	0.8415	0.7632
2	0.7716	0.7717	0.8421	0.8416	
4	0.7716	0.7717	0.8417	0.8418	
6	0.7715	0.7717	0.8413	0.8418	
8	0.7718	0.7719	0.8415	0.8514	
10	0.7720	0.7720	0.8411	0.8419	
12	0.7720	0.7720	0.8418	0.8415	
14	0.7720	0.7716	0.8413	0.8418	
16	0.7718	0.7720	0.8415	0.8417	
18	0.7718	0.7718	0.8411	0.8416	

Simulation parameters are as follows: population size = 2000; number of QTL = 200; heritability = 0.3; number of chromosomes = 5; and number of markers per chromosome = 40,000

### Application in Chinese Simmental beef cattle

#### Convergence diagnoses

Convergence diagnoses were conducted for residual variances as well as for a randomly selected number of SNP effects. Generally, the MCMC simulations of residual variances converged quickly, which primarily occurred within the first 1000 iterations, and the posterior modes were highly comparable among the various computing forms of the same genomic prediction models. Nevertheless, certain differences were observed in the posterior modes of the residual variances between different models (e.g., between TBM-BayesA and TBM-BayesCπ). These results were consistent with our observations in the simulation studies, with trivial differences in the estimated SNP effects and GEBVs (and hence GPAs) among various computing forms of the same statistical models caused by Monte Carlo errors and intrinsic differences observed between different statistical models. With the estimated residual variances of the four traits used as examples, the difference was the lowest for SLW, and the posterior mode of residual variance approached 0.16 with TBM-BayesA (Figure S1 in Additional file 1) and 0.18 with BayesCπ (Figure S2 in Additional file 1); the difference was largest for CBFT, and the posterior mode of the residual variance approached 0.19 (TBM-BayesA; Additional file 1: Figure S3) and 0.30 (TBM-BayesCπ; Additional file 1: Figure S4). Trace plots of the MCMC chains of residual variance for the remaining two traits (CW and ADG) are also provided in Additional file 1: Figures S5–S8. Trace plots of MCMC chains of selected SNP effects on SLW obtained by TMB-BayesA and TMB-BayesCπ are shown in Additional file 1: Figures S12 and S13, respectively. Evidently, these MCMC chains also all converged quickly within the first 1000 iterations.

#### Estimated heritabilities and GPAs

The estimated heritabilities for the four traits were within the range of previous reports [25, 26]. The differences may have been caused by differences in the genomic architectures of distinct breeds. In this Chinese Simmental beef population, CBFT had a smaller heritability compared with the other three traits. Consequently, the GPAs for CBFT were also lower (0.100 ~ 0.106) than those for the other three traits (0.202 ~ 0.271) (Table 4).

**Table 4:** Heritability estimates and predictive accuracies of four quantitative traits in a Chinese Simmental cattle population

Trait	h^2^	Correlations			Correlations divided by square root of heritability		
		GBLUP	TBM-BayesA	TBM-BayesCπ	GBLUP	TBM-BayesA	TBM-BayesCπ
CBFT	0.10	0.100	0.105	0.106	0.316	0.332	0.337
CW	0.45	0.266	0.271	0.268	0.397	0.404	0.399
SLW	0.24	0.202	0.213	0.215	0.413	0.435	0.440
ADG	0.47	0.214	0.204	0.206	0.312	0.297	0.301
Mean		0.196	0.198	0.199	0.360	0.367	0.369

*CBFT* carcass back fat thickness, *SLW* strip loin weight, *CW* carcass weight, *ADG* average daily gain, and *h*^*2*^ heritability

Nevertheless, the RGPAs were comparable among the four traits because this criterion assessed the GPAs relative to the square root of the heritability of each trait, with the latter reflecting the selection accuracy based on phenotypes and pedigree information. The RGPAs were also roughly comparable among the three models but with slight differences: TBM-BayesA and TBM-BayesCπ had a greater RGPA for CBFT, SLW, and CW but a lower RGPA for ADG; and TBM-BayesCπ had the greatest average RGPA for the four traits calculated across the three computational-statistical models. Again, these differences might not be based on computational differences but could be intrinsic to the differences in the data and statistical models.

## Discussion

### Parallel computing of Bayesian genomic prediction models: tunable burn-in versus fixed burn-in

Bayesian regression models are of high value for genomic prediction, although the complexity of computing of these models can be intensive [14], which is increasingly becoming the bottleneck in practical genomic selection programs. The challenges are found primarily in two aspects. First, genotype and phenotype data have been accumulating drastically in the past 10 years, and these “big data” are not managed efficiently because traditional data processing methods and tools are inadequate. Hence, high-performance computing (e.g., via parallel programming and computing strategies) is required to increase the computational efficiency and generate high computational throughputs for genomic selection. Nevertheless, the computing of Bayesian genomic prediction models is not parallelizable by the nature of the iterative algorithms, which poses the second and most likely greater challenge. Although Bayesian genomic prediction models can be calculated in parallel by running multiple MCMC chains of the same model, the speedup of computing heavily depends on the length of the burn-in period, which cannot be parallelized. Often, the length of burn-ins is set arbitrarily and thus can be too short or too long. When too short, the Markov chains are not converged, and the generated samples do not represent those drawn from the targeted posterior distributions. When too long, running a longer burn-in period after the convergence of Markov chains does not improve the accuracies of the posterior estimates of the model parameters [27, 28] but does consume more time than necessary. In the present study, we proposed a tunable, multiple-chain MCMC algorithm that is capable of automatically tuning an appropriate length of burn-ins, depending only on the actual status of MCMC convergence of the Bayesian statistical model. Our results showed that this tunable burn-in algorithm was effective and able to reduce the computing time remarkably compared with its counterpart with fixed burn-ins. In the present study, we used Gelman and Rubin’s convergence diagnostic method [15] to monitor the convergence state of the multiple-chain MCMC method. The shrink factor was calculated using posterior samples of the residual variance and a selected number of SNP effects from multiple chains of each model. When the multiple chains reached convergence, the burn-in period was terminated immediately, and the posterior samples generated afterward were collected and used for statistical inferences of the model parameters of interest, and they were subject to the frequency of thinning.

In the discussion that follows, we explain numerically how tunable burn-in MCMC could achieve greater speedups than fixed burn-in MCMC. Consider again the formula of the speedup ratio as in (10). Let N_burn-in_ = m and N_T_ = 5 m; in FBM-BayesA and FBM-BayesCπ, the speedup ratios are S(5) = 2.78 and S(20) = 4.16 when 5 and 20 cores are available for computing, respectively. Nevertheless, the maximal speedup ratio is $\underset{\mathrm{K}\to \infty }{\lim}\mathrm{S}\left(\mathrm{K}\right)=5$, regardless of how many cores are available for computing. Using our strategy, N_burn-in_ was adjusted to reduce unnecessary burn-in iterations based on the convergence diagnosis. If N_burn-in_ was shortened to m/2 by the convergence diagnosis, then the corresponding speedup ratios were doubled to S(5) = 3.84 and S(20) = 7.14. Furthermore, if N_burn-in_ was reduced to m/4, then the speedup ratios were quadrupled to S(5) = 4.76 and S(20) = 11.11. In the simulation studies, our results showed that TBM-BayesA (or TBM-BayesCπ) tended to a burn-in length that was half or even one-fourth as long as that of FBM-BayesA (or FBM-BayesCπ). This result demonstrated that the automatic tunable burn-in MCMC method effectively shortened the length of burn-ins compared with the fixed-burn-in MCMC; therefore, the tunable burn-in MCMC led to a greater speedup and greater parallel computing efficiency.

Our results showed that the use of tunable burn-in MCMC did not change the GPA as long as the MCMC chains converged. This conclusion is also supported by previous research [27, 28]. The GEBV is a critical concept in genomic prediction, and it was calculated as the sum of all SNP effects for each animal. Our results showed that the estimated SNP effects were highly analogous between the tunable burn-in MCMC and fixed burn-in MCMC used to calculate the same statistical model; however, the former had greater speedups than the latter. The differences were essentially caused by Monte Carlo errors. Because of such small differences in the estimated SNP effects among various forms of calculating the same genomic prediction model, the differences in the calculated GEBV for individual animals could mostly be ignored.

### Genomic predictions of Chinese Simmental beef cattle: a real application

The tunable burn-in MCMC and fixed burn-in MCMC methods were applied to genomic predictions of four quantitative traits in a Chinese Simmental beef population, and the GPAs obtained were also compared with those of the GBLUP. In the Chinese Simmental data set, the RGPAs were roughly comparable among the three models GBLUP, TBM-BayesA and TBM-BayesCπ because the RGPA assesses the GPA relative to the heritability of each trait in the present study. Without adjusting for the heritability differences of these traits, our results indicated that the GPA was higher for a trait with higher heritability than for traits with lower heritability. Nevertheless, both the GPA and RGPA showed noticeable differences among the different genomic prediction models, and these differences were not necessarily based on computational factors but could be intrinsic to the varied assumptions of these models. In reality, GPAs can vary with a number of factors, such as the size of the reference populations, heritability of the trait, density of the SNP panels, level of LD, and the statistical models used for prediction [2].

In this real application, our results supported that this tunable burn-in MCMC was effective and outperformed the fixed burn-in MCMC regarding speedup and parallel computing efficiency. The GPAs were comparable among the various computing forms of BayesA and BayesCπ, and these two models had greater GPAs for three of the four traits than GBLUP.

### Toward greater parallel computing efficiencies: what to consider?

Finally, several strategies deserve mention, such as adaptive MCMC algorithms [29, 30] and tempering [31, 32], which can further improve the convergence of multiple-chain MCMC. These strategies were not investigated in the present study but are worthy of investigation in future studies for further increasing parallel computing efficiency for genomic prediction. For example, Metropolis-coupled MCMC is a method that is related to simulated tempering and tempered transitions [31, 32] and simultaneously runs several different Markov chains governed by different (yet related) Markov chain transition probabilities. Occasionally, the algorithm “swaps” values between different chains, with the probability governed by the Metropolis algorithm to preserve the stationarity of the target distribution. Possibly, these swaps can speed up the convergence of the algorithm substantially. Craiu et al. proposed an ensemble of MCMC chains using the covariance of samples across all chains to adopt the proposed covariance for a set of Metropolis-Hastings chains [33]. Somewhat different from these multiple-chain methods, which use a synchronous exchange of samples to expedite convergence, Murray et al. mixed in an additional independent proposal that represents some hitherto best estimate or summary of the posterior and cooperative adapting across chains [34]. Therefore, a globally best estimate of the posterior is generated at any given step, and then this estimate is mixed as a remote component with whatever local proposal that a chain has adopted. This method does not preclude adaptive treatment or tempering of that local proposal but also permits a heterogeneous blend of remote proposals, thus allowing the ensemble of chains to mix well.

## Conclusions

An automatically tunable burn-in MCMC method for calculating Bayesian genomic prediction models was proposed and manifested using BayesA and BayesCπ models. Our results from the simulation study showed a better speedup in computing with tunable burn-in MCMC than with fixed burn-in MCMC. However, the estimated SNPs and GPAs were highly comparable regardless of the various forms of parallel computing when the same Bayesian genomic prediction model was used. In a Chinese Simmental beef population, the average GPAs for four quantitative traits obtained by the tunable burn-in BayesA and BayesCπ models were better than those obtained by the GBLUP, and although these differences may have been caused by computational factors, they might also have been attributable to intrinsic differences in the statistical model assumptions. The proposed tunable burn-in strategy for running parallel (i.e., multiple-chain) MCMC can lead to dramatically increased computational efficiency and is applicable to the computing of all complex Bayesian models, either sequentially or in parallel.

## Additional file

Additional file 1: Figure S1. Trace plots of posterior samples of residual variance from TBM-BayesA (16 chains) for SW of the first 2000 iterations. **Figure S2.** Trace plots of posterior samples of residual variance from TBM-BayesCπ (16 chains) for SW of the first 2000 iterations. **Figure S3.** Trace plots of posterior samples of residual variance from TBM-BayesA (16 chains) for CBFT of the first 1800 iterations. **Figure S4.** Trace plots of posterior samples of residual variance from TBM-BayesCπ (16 chains) for CBFT of the first 2500 iterations. **Figure S5.** Trace plots of posterior samples of residual variance from TBM-BayesA (16 chains) for CW of the first 2000 iterations. **Figure S6.** Trace plots of posterior samples of residual variance from TBM-BayesCπ (16 chains) for CW of the first 2000 iterations. **Figure S7.** Trace plots of posterior samples of residual variance from TBM-BayesA (16 chains) for ADG of the first 1400 iterations. **Figure S8.** Trace plots of posterior samples of residual variance from TBM-BayesCπ (16 chains) for ADG of the first 1400 iterations. **Figure S9.** Plot of running time in scenario1.FBM-BayesA: Fixed burn-in multiple chains parallel BayesA, TBM-BayesA: Tunable burn-in multiple chains parallel BayesA, FBM-BayesCπ: Fixed burn-in multiple chains parallel BayesCπ, TBM-BayesCπ: Tunable burn-in multiple chains parallel BayesCπ. One chain is equivalent to sequential genomic selection. **Figure S10.** Plot of running time in scenario 2. **Figure S11.** Plot of running time in scenario 3 **Figure S12.** Convergence of TBM-BayesA for SW. Iteration: 50,000, initial Burn-in:10,000, threshold:0.001. **Figure S13.** Convergence of TBM-BayesCπ for SW. Iteration: 50,000, initial Burn-in:10,000, threshold:0.001. **Table S1.** Running time using five genomic prediction approaches in scenario 1. **Table S2.** Running time using five genomic prediction approaches in scenario 2. **Table S3.** Running time using five genomic prediction approaches in scenario 3. (ZIP 405 kb)

## Abbreviations

ADG: Average daily gain
CBFT: Carcass back fat thickness
CW: Carcass weight
FBM: Fixed burn-in, multiple-chain
GBLUP: Genomic Best Linear Unbiased Prediction
GEBVs: Genomic estimated breeding values
GPA: Genomic prediction accuracy
h^2^: heritability
MCMC: Markov chain Monte Carlo
MPI: Message Passing Interface
QTL: Quantitative trait locus
REML: Restricted maximum likelihood
RGPA: Relative genomic prediction accuracy
SLW: Strip loin weight
TBM: Tunable burn-in, multiple-chain
