# Supplementary figures and images for: Fast genomic prediction of breeding values using parallel Markov chain Monte Carlo with convergence diagnosis
Source: BMC Bioinformatics. 2018 Jan 3;19:3. doi: 10.1186/s12859-017-2003-3 (PMC5751823; doi:10.1186/s12859-017-2003-3)

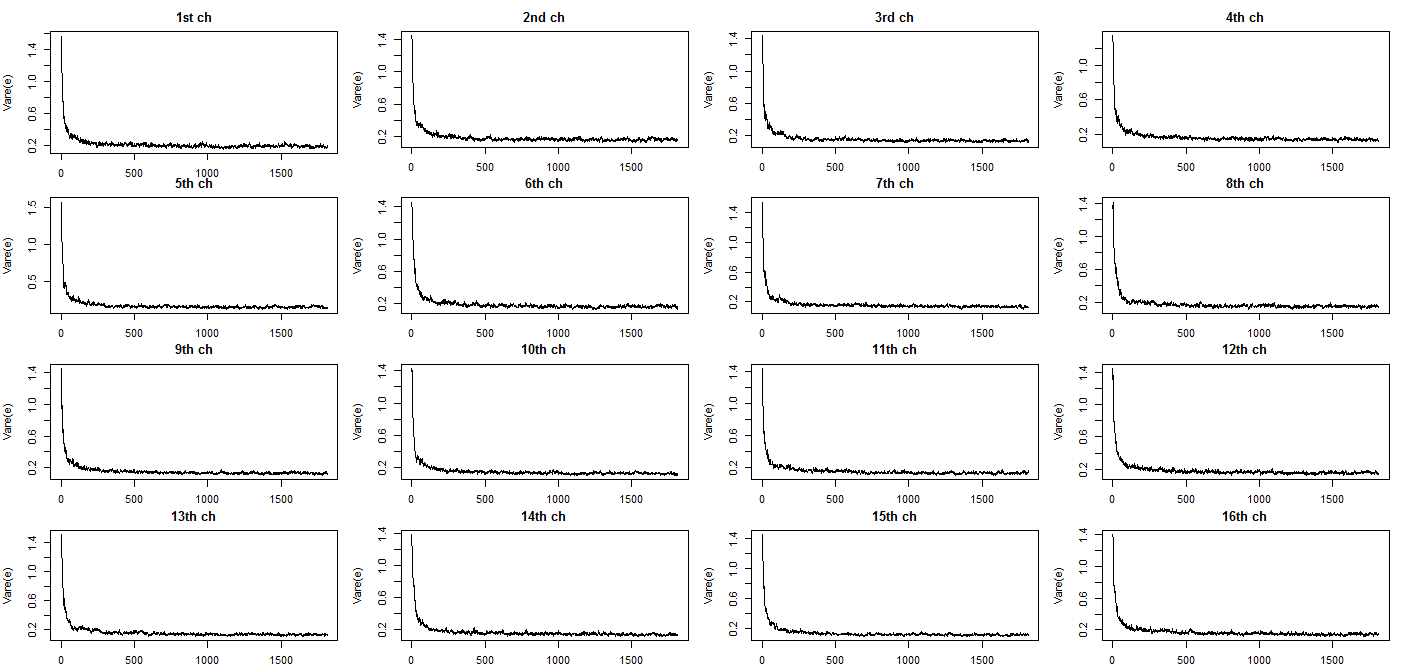

Supplement: Additional file 1: Figure S1. — Trace plots of posterior samples of residual variance from TBM-BayesA (16 chains) for SW of the first 2000 iterations. Figure S2. Trace plots of posterior samples of residual variance from TBM-BayesCπ (16 chains) for SW of the first 2000 iterations. Figure S3. Trace plots of posterior samples of residual variance from TBM-BayesA (16 chains) for CBFT of the first 1800 iterations. Figure S4. Trace plots of posterior samples of residual variance from TBM-BayesCπ (16 chains) for CBFT of the first 2500 iterations. Figure S5. Trace plots of posterior samples of residual variance from TBM-BayesA (16 chains) for CW of the first 2000 iterations. Figure S6. Trace plots of posterior samples of residual variance from TBM-BayesCπ (16 chains) for CW of the first 2000 iterations. Figure S7. Trace plots of posterior samples of residual variance from TBM-BayesA (16 chains) for ADG of the first 1400 iterations. Figure S8. Trace plots of posterior samples of residual variance from TBM-BayesCπ (16 chains) for ADG of the first 1400 iterations. Figure S9. Plot of running time in scenario1.FBM-BayesA: Fixed burn-in multiple chains parallel BayesA, TBM-BayesA: Tunable burn-in multiple chains parallel BayesA, FBM-BayesCπ: Fixed burn-in multiple chains parallel BayesCπ, TBM-BayesCπ: Tunable burn-in multiple chains parallel BayesCπ. One chain is equivalent to sequential genomic selection. Figure S10. Plot of running time in scenario 2. Figure S11. Plot of running time in scenario 3 Figure S12. Convergence of TBM-BayesA for SW. Iteration: 50,000, initial Burn-in:10,000, threshold:0.001. Figure S13. Convergence of TBM-BayesCπ for SW. Iteration: 50,000, initial Burn-in:10,000, threshold:0.001. Table S1. Running time using five genomic prediction approaches in scenario 1. Table S2. Running time using five genomic prediction approaches in scenario 2. Table S3. Running time using five genomic prediction approaches in scenario 3. (ZIP 405 kb) [file 12859_2017_2003_MOESM1_ESM.zip › Supplementary file/Fig. S1 Residual variance from TBM-BayesA for SW.png]

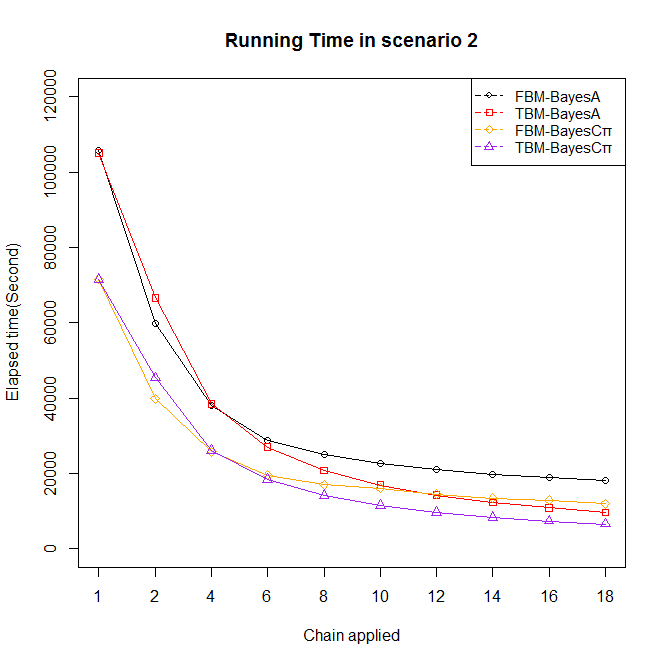

Supplement: Additional file 1: Figure S1. — Trace plots of posterior samples of residual variance from TBM-BayesA (16 chains) for SW of the first 2000 iterations. Figure S2. Trace plots of posterior samples of residual variance from TBM-BayesCπ (16 chains) for SW of the first 2000 iterations. Figure S3. Trace plots of posterior samples of residual variance from TBM-BayesA (16 chains) for CBFT of the first 1800 iterations. Figure S4. Trace plots of posterior samples of residual variance from TBM-BayesCπ (16 chains) for CBFT of the first 2500 iterations. Figure S5. Trace plots of posterior samples of residual variance from TBM-BayesA (16 chains) for CW of the first 2000 iterations. Figure S6. Trace plots of posterior samples of residual variance from TBM-BayesCπ (16 chains) for CW of the first 2000 iterations. Figure S7. Trace plots of posterior samples of residual variance from TBM-BayesA (16 chains) for ADG of the first 1400 iterations. Figure S8. Trace plots of posterior samples of residual variance from TBM-BayesCπ (16 chains) for ADG of the first 1400 iterations. Figure S9. Plot of running time in scenario1.FBM-BayesA: Fixed burn-in multiple chains parallel BayesA, TBM-BayesA: Tunable burn-in multiple chains parallel BayesA, FBM-BayesCπ: Fixed burn-in multiple chains parallel BayesCπ, TBM-BayesCπ: Tunable burn-in multiple chains parallel BayesCπ. One chain is equivalent to sequential genomic selection. Figure S10. Plot of running time in scenario 2. Figure S11. Plot of running time in scenario 3 Figure S12. Convergence of TBM-BayesA for SW. Iteration: 50,000, initial Burn-in:10,000, threshold:0.001. Figure S13. Convergence of TBM-BayesCπ for SW. Iteration: 50,000, initial Burn-in:10,000, threshold:0.001. Table S1. Running time using five genomic prediction approaches in scenario 1. Table S2. Running time using five genomic prediction approaches in scenario 2. Table S3. Running time using five genomic prediction approaches in scenario 3. (ZIP 405 kb) [file 12859_2017_2003_MOESM1_ESM.zip › Supplementary file/Fig. S10 Runtime in scenario 2.png]

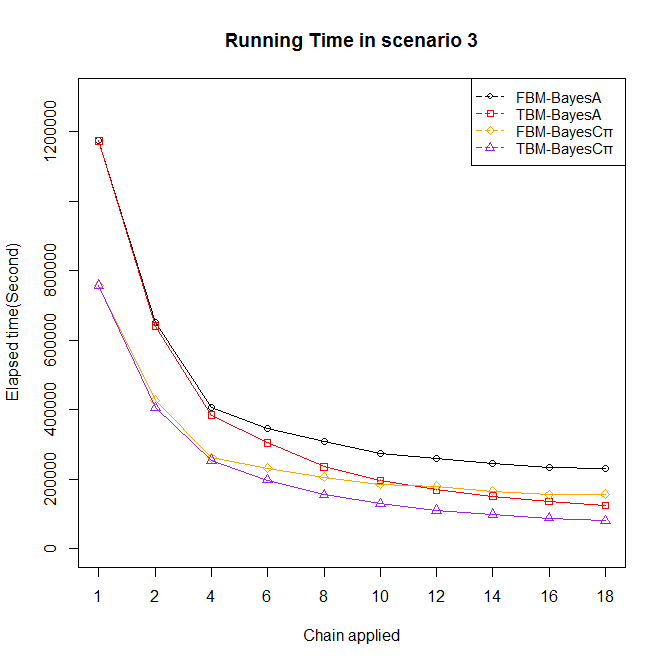

Supplement: Additional file 1: Figure S1. — Trace plots of posterior samples of residual variance from TBM-BayesA (16 chains) for SW of the first 2000 iterations. Figure S2. Trace plots of posterior samples of residual variance from TBM-BayesCπ (16 chains) for SW of the first 2000 iterations. Figure S3. Trace plots of posterior samples of residual variance from TBM-BayesA (16 chains) for CBFT of the first 1800 iterations. Figure S4. Trace plots of posterior samples of residual variance from TBM-BayesCπ (16 chains) for CBFT of the first 2500 iterations. Figure S5. Trace plots of posterior samples of residual variance from TBM-BayesA (16 chains) for CW of the first 2000 iterations. Figure S6. Trace plots of posterior samples of residual variance from TBM-BayesCπ (16 chains) for CW of the first 2000 iterations. Figure S7. Trace plots of posterior samples of residual variance from TBM-BayesA (16 chains) for ADG of the first 1400 iterations. Figure S8. Trace plots of posterior samples of residual variance from TBM-BayesCπ (16 chains) for ADG of the first 1400 iterations. Figure S9. Plot of running time in scenario1.FBM-BayesA: Fixed burn-in multiple chains parallel BayesA, TBM-BayesA: Tunable burn-in multiple chains parallel BayesA, FBM-BayesCπ: Fixed burn-in multiple chains parallel BayesCπ, TBM-BayesCπ: Tunable burn-in multiple chains parallel BayesCπ. One chain is equivalent to sequential genomic selection. Figure S10. Plot of running time in scenario 2. Figure S11. Plot of running time in scenario 3 Figure S12. Convergence of TBM-BayesA for SW. Iteration: 50,000, initial Burn-in:10,000, threshold:0.001. Figure S13. Convergence of TBM-BayesCπ for SW. Iteration: 50,000, initial Burn-in:10,000, threshold:0.001. Table S1. Running time using five genomic prediction approaches in scenario 1. Table S2. Running time using five genomic prediction approaches in scenario 2. Table S3. Running time using five genomic prediction approaches in scenario 3. (ZIP 405 kb) [file 12859_2017_2003_MOESM1_ESM.zip › Supplementary file/Fig. S11 Runtime in scenario 3.png]

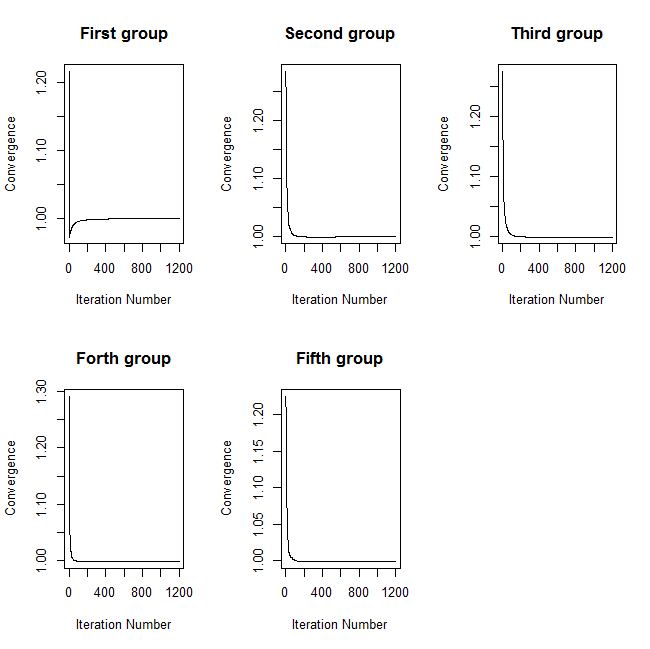

Supplement: Additional file 1: Figure S1. — Trace plots of posterior samples of residual variance from TBM-BayesA (16 chains) for SW of the first 2000 iterations. Figure S2. Trace plots of posterior samples of residual variance from TBM-BayesCπ (16 chains) for SW of the first 2000 iterations. Figure S3. Trace plots of posterior samples of residual variance from TBM-BayesA (16 chains) for CBFT of the first 1800 iterations. Figure S4. Trace plots of posterior samples of residual variance from TBM-BayesCπ (16 chains) for CBFT of the first 2500 iterations. Figure S5. Trace plots of posterior samples of residual variance from TBM-BayesA (16 chains) for CW of the first 2000 iterations. Figure S6. Trace plots of posterior samples of residual variance from TBM-BayesCπ (16 chains) for CW of the first 2000 iterations. Figure S7. Trace plots of posterior samples of residual variance from TBM-BayesA (16 chains) for ADG of the first 1400 iterations. Figure S8. Trace plots of posterior samples of residual variance from TBM-BayesCπ (16 chains) for ADG of the first 1400 iterations. Figure S9. Plot of running time in scenario1.FBM-BayesA: Fixed burn-in multiple chains parallel BayesA, TBM-BayesA: Tunable burn-in multiple chains parallel BayesA, FBM-BayesCπ: Fixed burn-in multiple chains parallel BayesCπ, TBM-BayesCπ: Tunable burn-in multiple chains parallel BayesCπ. One chain is equivalent to sequential genomic selection. Figure S10. Plot of running time in scenario 2. Figure S11. Plot of running time in scenario 3 Figure S12. Convergence of TBM-BayesA for SW. Iteration: 50,000, initial Burn-in:10,000, threshold:0.001. Figure S13. Convergence of TBM-BayesCπ for SW. Iteration: 50,000, initial Burn-in:10,000, threshold:0.001. Table S1. Running time using five genomic prediction approaches in scenario 1. Table S2. Running time using five genomic prediction approaches in scenario 2. Table S3. Running time using five genomic prediction approaches in scenario 3. (ZIP 405 kb) [file 12859_2017_2003_MOESM1_ESM.zip › Supplementary file/Fig. S12 Convergence of TBM-BayesA for SW.png]

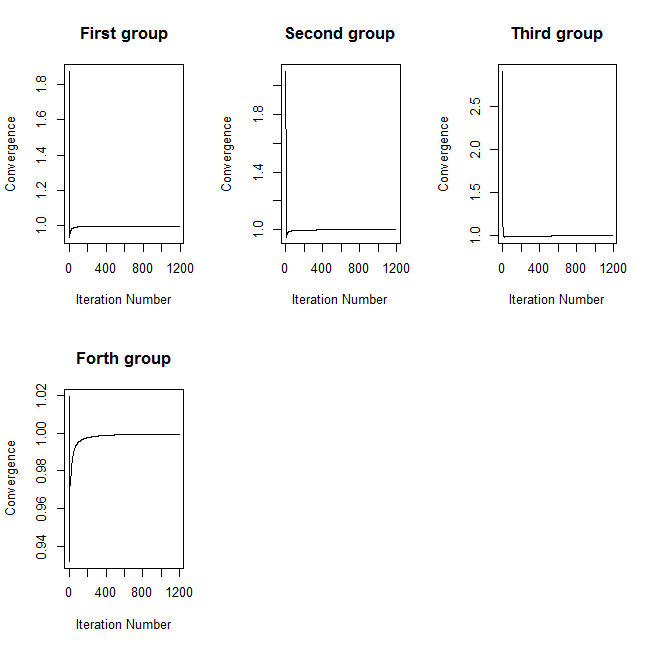

Supplement: Additional file 1: Figure S1. — Trace plots of posterior samples of residual variance from TBM-BayesA (16 chains) for SW of the first 2000 iterations. Figure S2. Trace plots of posterior samples of residual variance from TBM-BayesCπ (16 chains) for SW of the first 2000 iterations. Figure S3. Trace plots of posterior samples of residual variance from TBM-BayesA (16 chains) for CBFT of the first 1800 iterations. Figure S4. Trace plots of posterior samples of residual variance from TBM-BayesCπ (16 chains) for CBFT of the first 2500 iterations. Figure S5. Trace plots of posterior samples of residual variance from TBM-BayesA (16 chains) for CW of the first 2000 iterations. Figure S6. Trace plots of posterior samples of residual variance from TBM-BayesCπ (16 chains) for CW of the first 2000 iterations. Figure S7. Trace plots of posterior samples of residual variance from TBM-BayesA (16 chains) for ADG of the first 1400 iterations. Figure S8. Trace plots of posterior samples of residual variance from TBM-BayesCπ (16 chains) for ADG of the first 1400 iterations. Figure S9. Plot of running time in scenario1.FBM-BayesA: Fixed burn-in multiple chains parallel BayesA, TBM-BayesA: Tunable burn-in multiple chains parallel BayesA, FBM-BayesCπ: Fixed burn-in multiple chains parallel BayesCπ, TBM-BayesCπ: Tunable burn-in multiple chains parallel BayesCπ. One chain is equivalent to sequential genomic selection. Figure S10. Plot of running time in scenario 2. Figure S11. Plot of running time in scenario 3 Figure S12. Convergence of TBM-BayesA for SW. Iteration: 50,000, initial Burn-in:10,000, threshold:0.001. Figure S13. Convergence of TBM-BayesCπ for SW. Iteration: 50,000, initial Burn-in:10,000, threshold:0.001. Table S1. Running time using five genomic prediction approaches in scenario 1. Table S2. Running time using five genomic prediction approaches in scenario 2. Table S3. Running time using five genomic prediction approaches in scenario 3. (ZIP 405 kb) [file 12859_2017_2003_MOESM1_ESM.zip › Supplementary file/Fig. S13 Convergence of TBM-BayesCa- fo SW.png]

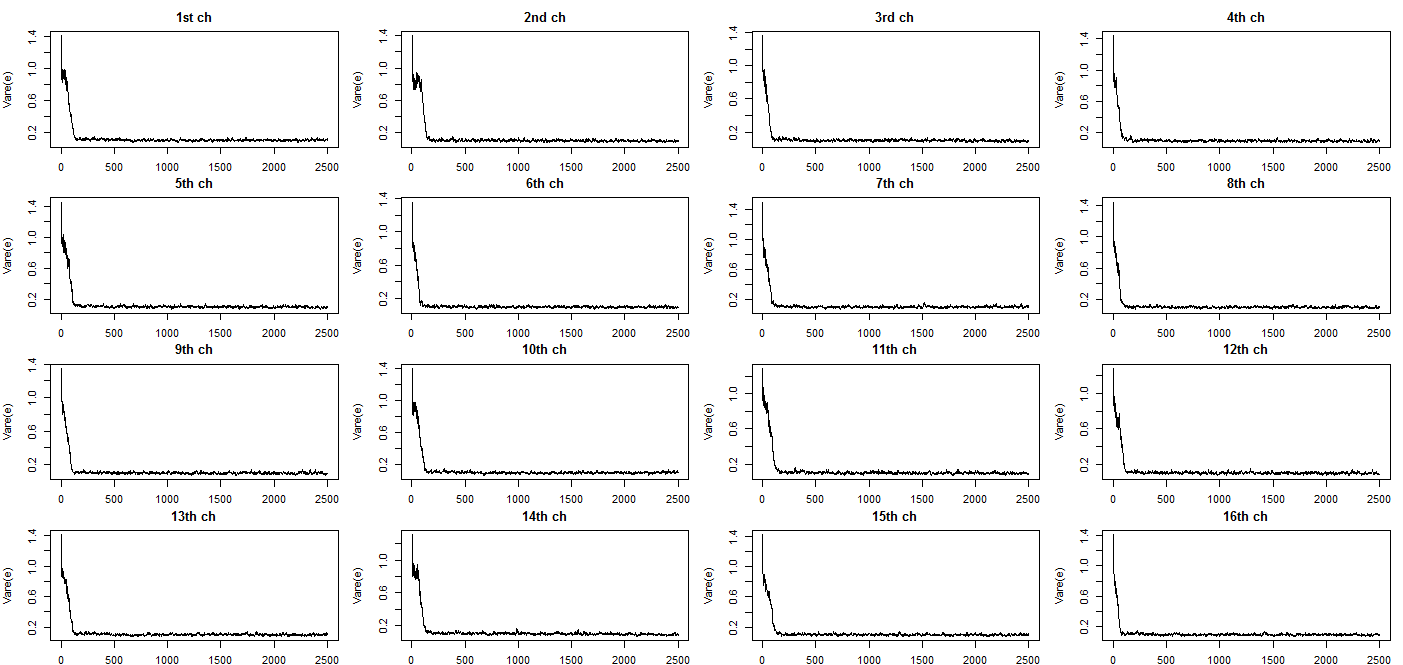

Supplement: Additional file 1: Figure S1. — Trace plots of posterior samples of residual variance from TBM-BayesA (16 chains) for SW of the first 2000 iterations. Figure S2. Trace plots of posterior samples of residual variance from TBM-BayesCπ (16 chains) for SW of the first 2000 iterations. Figure S3. Trace plots of posterior samples of residual variance from TBM-BayesA (16 chains) for CBFT of the first 1800 iterations. Figure S4. Trace plots of posterior samples of residual variance from TBM-BayesCπ (16 chains) for CBFT of the first 2500 iterations. Figure S5. Trace plots of posterior samples of residual variance from TBM-BayesA (16 chains) for CW of the first 2000 iterations. Figure S6. Trace plots of posterior samples of residual variance from TBM-BayesCπ (16 chains) for CW of the first 2000 iterations. Figure S7. Trace plots of posterior samples of residual variance from TBM-BayesA (16 chains) for ADG of the first 1400 iterations. Figure S8. Trace plots of posterior samples of residual variance from TBM-BayesCπ (16 chains) for ADG of the first 1400 iterations. Figure S9. Plot of running time in scenario1.FBM-BayesA: Fixed burn-in multiple chains parallel BayesA, TBM-BayesA: Tunable burn-in multiple chains parallel BayesA, FBM-BayesCπ: Fixed burn-in multiple chains parallel BayesCπ, TBM-BayesCπ: Tunable burn-in multiple chains parallel BayesCπ. One chain is equivalent to sequential genomic selection. Figure S10. Plot of running time in scenario 2. Figure S11. Plot of running time in scenario 3 Figure S12. Convergence of TBM-BayesA for SW. Iteration: 50,000, initial Burn-in:10,000, threshold:0.001. Figure S13. Convergence of TBM-BayesCπ for SW. Iteration: 50,000, initial Burn-in:10,000, threshold:0.001. Table S1. Running time using five genomic prediction approaches in scenario 1. Table S2. Running time using five genomic prediction approaches in scenario 2. Table S3. Running time using five genomic prediction approaches in scenario 3. (ZIP 405 kb) [file 12859_2017_2003_MOESM1_ESM.zip › Supplementary file/Fig. S2 Residual variance from TBM-BayesCa- for SW.png]

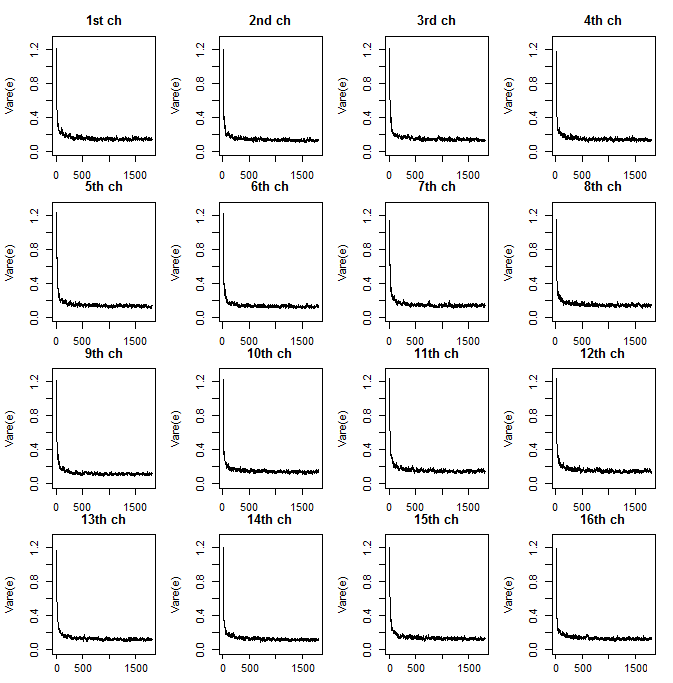

Supplement: Additional file 1: Figure S1. — Trace plots of posterior samples of residual variance from TBM-BayesA (16 chains) for SW of the first 2000 iterations. Figure S2. Trace plots of posterior samples of residual variance from TBM-BayesCπ (16 chains) for SW of the first 2000 iterations. Figure S3. Trace plots of posterior samples of residual variance from TBM-BayesA (16 chains) for CBFT of the first 1800 iterations. Figure S4. Trace plots of posterior samples of residual variance from TBM-BayesCπ (16 chains) for CBFT of the first 2500 iterations. Figure S5. Trace plots of posterior samples of residual variance from TBM-BayesA (16 chains) for CW of the first 2000 iterations. Figure S6. Trace plots of posterior samples of residual variance from TBM-BayesCπ (16 chains) for CW of the first 2000 iterations. Figure S7. Trace plots of posterior samples of residual variance from TBM-BayesA (16 chains) for ADG of the first 1400 iterations. Figure S8. Trace plots of posterior samples of residual variance from TBM-BayesCπ (16 chains) for ADG of the first 1400 iterations. Figure S9. Plot of running time in scenario1.FBM-BayesA: Fixed burn-in multiple chains parallel BayesA, TBM-BayesA: Tunable burn-in multiple chains parallel BayesA, FBM-BayesCπ: Fixed burn-in multiple chains parallel BayesCπ, TBM-BayesCπ: Tunable burn-in multiple chains parallel BayesCπ. One chain is equivalent to sequential genomic selection. Figure S10. Plot of running time in scenario 2. Figure S11. Plot of running time in scenario 3 Figure S12. Convergence of TBM-BayesA for SW. Iteration: 50,000, initial Burn-in:10,000, threshold:0.001. Figure S13. Convergence of TBM-BayesCπ for SW. Iteration: 50,000, initial Burn-in:10,000, threshold:0.001. Table S1. Running time using five genomic prediction approaches in scenario 1. Table S2. Running time using five genomic prediction approaches in scenario 2. Table S3. Running time using five genomic prediction approaches in scenario 3. (ZIP 405 kb) [file 12859_2017_2003_MOESM1_ESM.zip › Supplementary file/Fig. S3 Residual variance from TBM-BayesA for CBFT.png]

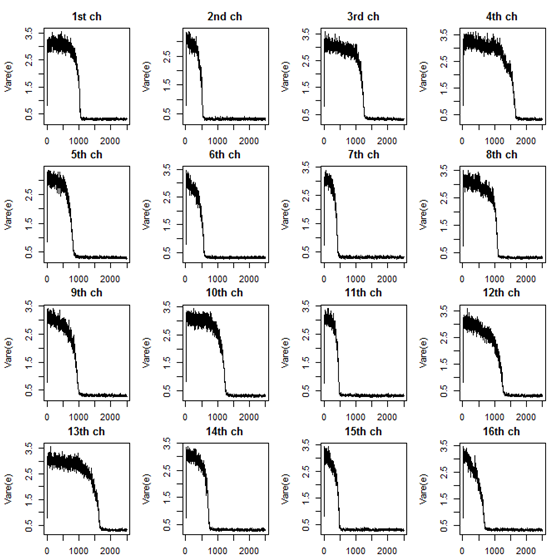

Supplement: Additional file 1: Figure S1. — Trace plots of posterior samples of residual variance from TBM-BayesA (16 chains) for SW of the first 2000 iterations. Figure S2. Trace plots of posterior samples of residual variance from TBM-BayesCπ (16 chains) for SW of the first 2000 iterations. Figure S3. Trace plots of posterior samples of residual variance from TBM-BayesA (16 chains) for CBFT of the first 1800 iterations. Figure S4. Trace plots of posterior samples of residual variance from TBM-BayesCπ (16 chains) for CBFT of the first 2500 iterations. Figure S5. Trace plots of posterior samples of residual variance from TBM-BayesA (16 chains) for CW of the first 2000 iterations. Figure S6. Trace plots of posterior samples of residual variance from TBM-BayesCπ (16 chains) for CW of the first 2000 iterations. Figure S7. Trace plots of posterior samples of residual variance from TBM-BayesA (16 chains) for ADG of the first 1400 iterations. Figure S8. Trace plots of posterior samples of residual variance from TBM-BayesCπ (16 chains) for ADG of the first 1400 iterations. Figure S9. Plot of running time in scenario1.FBM-BayesA: Fixed burn-in multiple chains parallel BayesA, TBM-BayesA: Tunable burn-in multiple chains parallel BayesA, FBM-BayesCπ: Fixed burn-in multiple chains parallel BayesCπ, TBM-BayesCπ: Tunable burn-in multiple chains parallel BayesCπ. One chain is equivalent to sequential genomic selection. Figure S10. Plot of running time in scenario 2. Figure S11. Plot of running time in scenario 3 Figure S12. Convergence of TBM-BayesA for SW. Iteration: 50,000, initial Burn-in:10,000, threshold:0.001. Figure S13. Convergence of TBM-BayesCπ for SW. Iteration: 50,000, initial Burn-in:10,000, threshold:0.001. Table S1. Running time using five genomic prediction approaches in scenario 1. Table S2. Running time using five genomic prediction approaches in scenario 2. Table S3. Running time using five genomic prediction approaches in scenario 3. (ZIP 405 kb) [file 12859_2017_2003_MOESM1_ESM.zip › Supplementary file/Fig. S4 Residual variance from TBM-BayesCa- for CBFT.png]

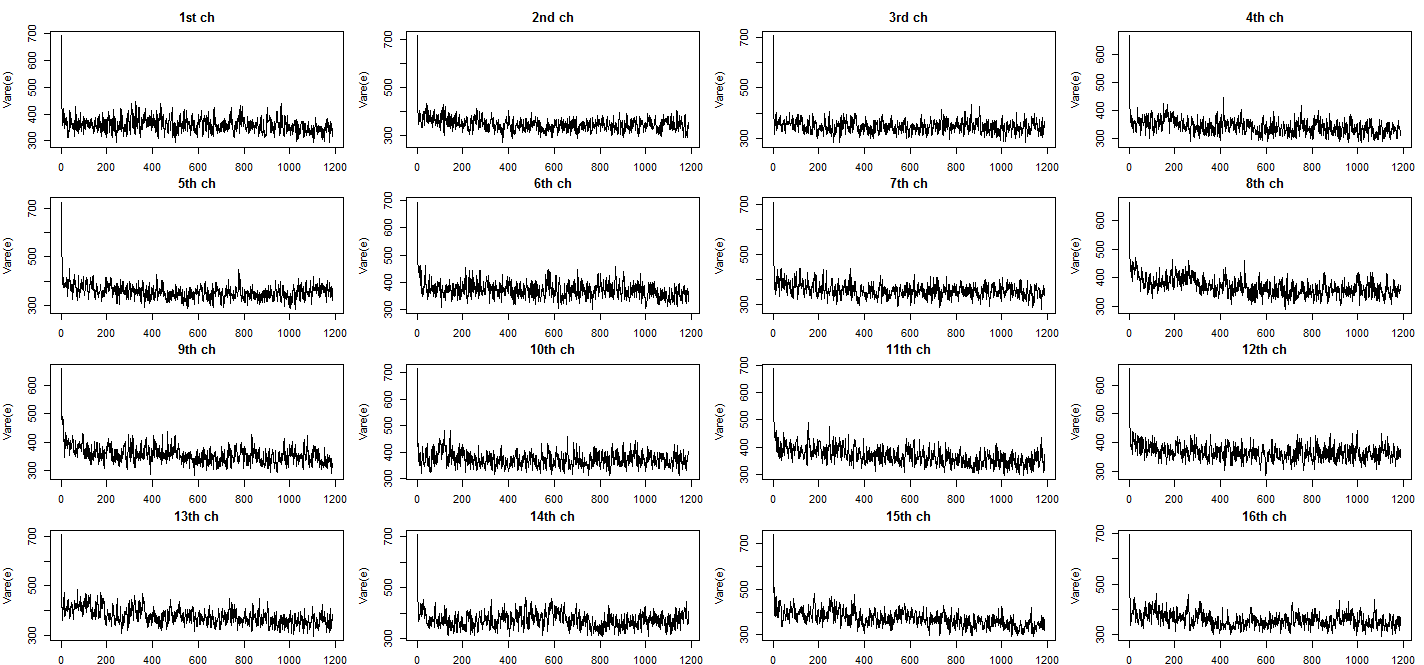

Supplement: Additional file 1: Figure S1. — Trace plots of posterior samples of residual variance from TBM-BayesA (16 chains) for SW of the first 2000 iterations. Figure S2. Trace plots of posterior samples of residual variance from TBM-BayesCπ (16 chains) for SW of the first 2000 iterations. Figure S3. Trace plots of posterior samples of residual variance from TBM-BayesA (16 chains) for CBFT of the first 1800 iterations. Figure S4. Trace plots of posterior samples of residual variance from TBM-BayesCπ (16 chains) for CBFT of the first 2500 iterations. Figure S5. Trace plots of posterior samples of residual variance from TBM-BayesA (16 chains) for CW of the first 2000 iterations. Figure S6. Trace plots of posterior samples of residual variance from TBM-BayesCπ (16 chains) for CW of the first 2000 iterations. Figure S7. Trace plots of posterior samples of residual variance from TBM-BayesA (16 chains) for ADG of the first 1400 iterations. Figure S8. Trace plots of posterior samples of residual variance from TBM-BayesCπ (16 chains) for ADG of the first 1400 iterations. Figure S9. Plot of running time in scenario1.FBM-BayesA: Fixed burn-in multiple chains parallel BayesA, TBM-BayesA: Tunable burn-in multiple chains parallel BayesA, FBM-BayesCπ: Fixed burn-in multiple chains parallel BayesCπ, TBM-BayesCπ: Tunable burn-in multiple chains parallel BayesCπ. One chain is equivalent to sequential genomic selection. Figure S10. Plot of running time in scenario 2. Figure S11. Plot of running time in scenario 3 Figure S12. Convergence of TBM-BayesA for SW. Iteration: 50,000, initial Burn-in:10,000, threshold:0.001. Figure S13. Convergence of TBM-BayesCπ for SW. Iteration: 50,000, initial Burn-in:10,000, threshold:0.001. Table S1. Running time using five genomic prediction approaches in scenario 1. Table S2. Running time using five genomic prediction approaches in scenario 2. Table S3. Running time using five genomic prediction approaches in scenario 3. (ZIP 405 kb) [file 12859_2017_2003_MOESM1_ESM.zip › Supplementary file/Fig. S5 Residual variance from TBM-BayesA for CW.png]

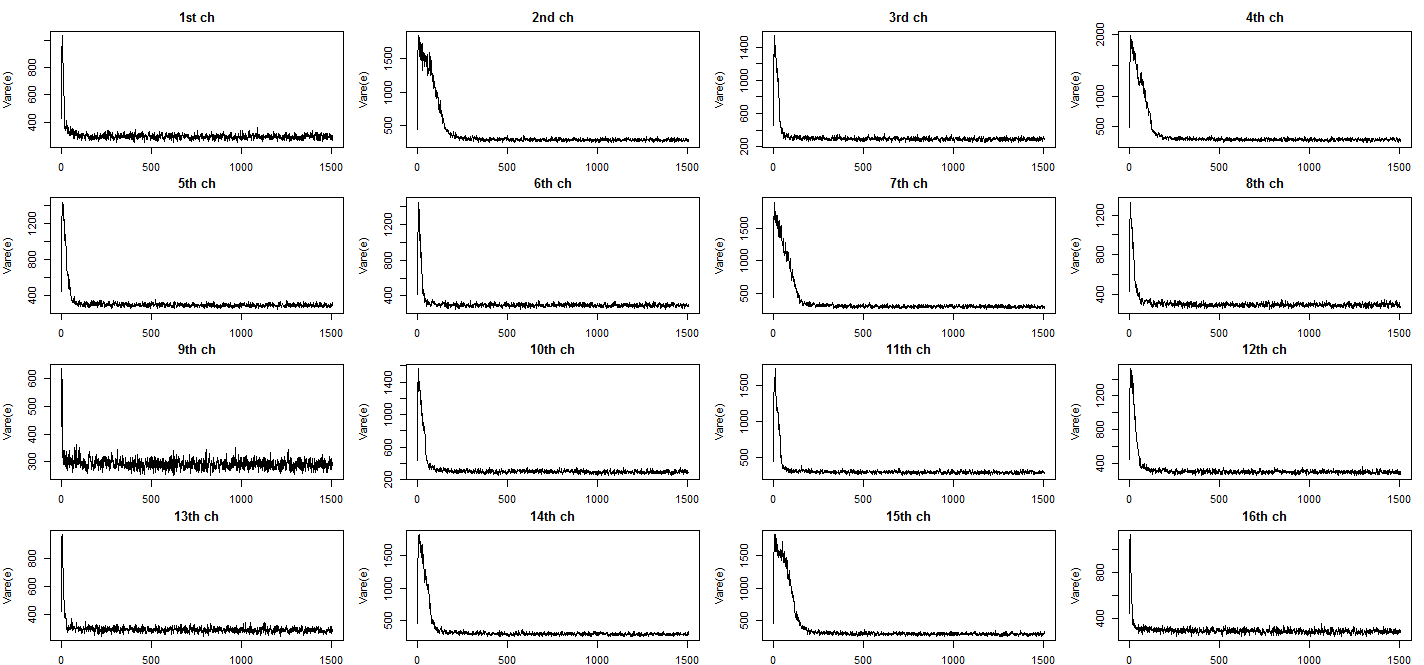

Supplement: Additional file 1: Figure S1. — Trace plots of posterior samples of residual variance from TBM-BayesA (16 chains) for SW of the first 2000 iterations. Figure S2. Trace plots of posterior samples of residual variance from TBM-BayesCπ (16 chains) for SW of the first 2000 iterations. Figure S3. Trace plots of posterior samples of residual variance from TBM-BayesA (16 chains) for CBFT of the first 1800 iterations. Figure S4. Trace plots of posterior samples of residual variance from TBM-BayesCπ (16 chains) for CBFT of the first 2500 iterations. Figure S5. Trace plots of posterior samples of residual variance from TBM-BayesA (16 chains) for CW of the first 2000 iterations. Figure S6. Trace plots of posterior samples of residual variance from TBM-BayesCπ (16 chains) for CW of the first 2000 iterations. Figure S7. Trace plots of posterior samples of residual variance from TBM-BayesA (16 chains) for ADG of the first 1400 iterations. Figure S8. Trace plots of posterior samples of residual variance from TBM-BayesCπ (16 chains) for ADG of the first 1400 iterations. Figure S9. Plot of running time in scenario1.FBM-BayesA: Fixed burn-in multiple chains parallel BayesA, TBM-BayesA: Tunable burn-in multiple chains parallel BayesA, FBM-BayesCπ: Fixed burn-in multiple chains parallel BayesCπ, TBM-BayesCπ: Tunable burn-in multiple chains parallel BayesCπ. One chain is equivalent to sequential genomic selection. Figure S10. Plot of running time in scenario 2. Figure S11. Plot of running time in scenario 3 Figure S12. Convergence of TBM-BayesA for SW. Iteration: 50,000, initial Burn-in:10,000, threshold:0.001. Figure S13. Convergence of TBM-BayesCπ for SW. Iteration: 50,000, initial Burn-in:10,000, threshold:0.001. Table S1. Running time using five genomic prediction approaches in scenario 1. Table S2. Running time using five genomic prediction approaches in scenario 2. Table S3. Running time using five genomic prediction approaches in scenario 3. (ZIP 405 kb) [file 12859_2017_2003_MOESM1_ESM.zip › Supplementary file/Fig. S6 Residual variance from TBM-BayesCa- for CW.png]

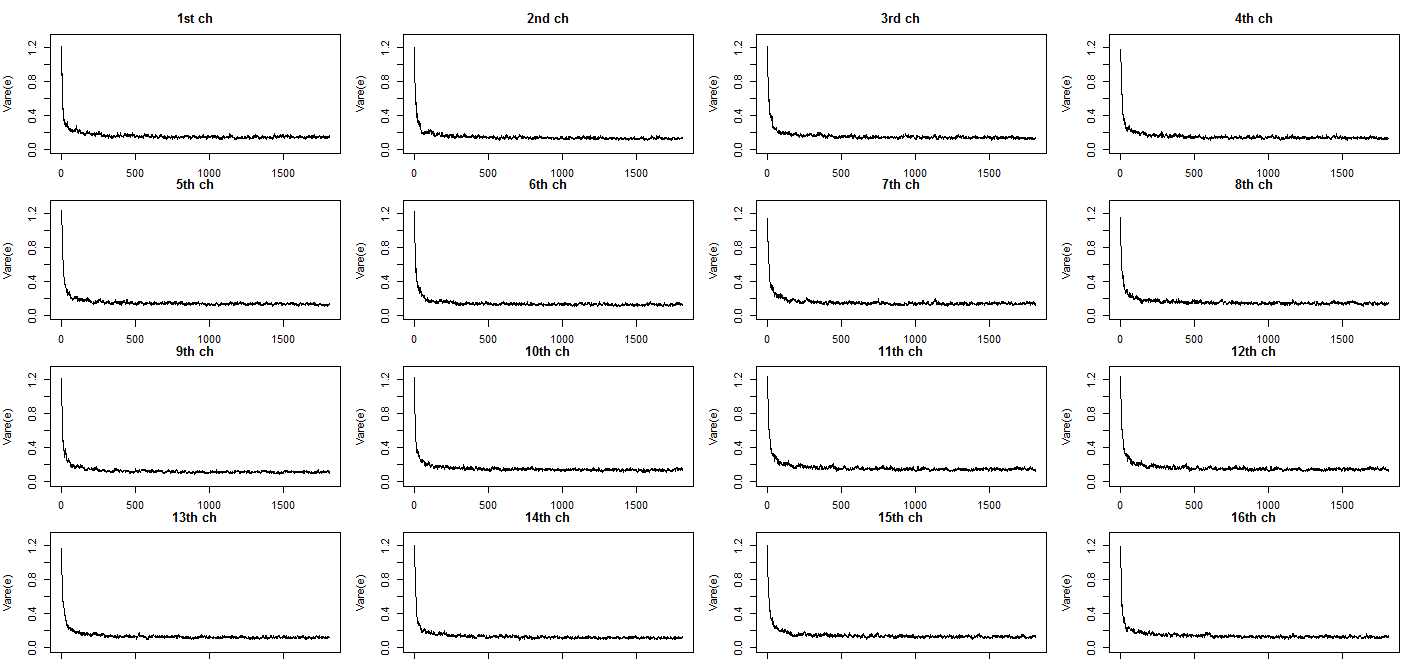

Supplement: Additional file 1: Figure S1. — Trace plots of posterior samples of residual variance from TBM-BayesA (16 chains) for SW of the first 2000 iterations. Figure S2. Trace plots of posterior samples of residual variance from TBM-BayesCπ (16 chains) for SW of the first 2000 iterations. Figure S3. Trace plots of posterior samples of residual variance from TBM-BayesA (16 chains) for CBFT of the first 1800 iterations. Figure S4. Trace plots of posterior samples of residual variance from TBM-BayesCπ (16 chains) for CBFT of the first 2500 iterations. Figure S5. Trace plots of posterior samples of residual variance from TBM-BayesA (16 chains) for CW of the first 2000 iterations. Figure S6. Trace plots of posterior samples of residual variance from TBM-BayesCπ (16 chains) for CW of the first 2000 iterations. Figure S7. Trace plots of posterior samples of residual variance from TBM-BayesA (16 chains) for ADG of the first 1400 iterations. Figure S8. Trace plots of posterior samples of residual variance from TBM-BayesCπ (16 chains) for ADG of the first 1400 iterations. Figure S9. Plot of running time in scenario1.FBM-BayesA: Fixed burn-in multiple chains parallel BayesA, TBM-BayesA: Tunable burn-in multiple chains parallel BayesA, FBM-BayesCπ: Fixed burn-in multiple chains parallel BayesCπ, TBM-BayesCπ: Tunable burn-in multiple chains parallel BayesCπ. One chain is equivalent to sequential genomic selection. Figure S10. Plot of running time in scenario 2. Figure S11. Plot of running time in scenario 3 Figure S12. Convergence of TBM-BayesA for SW. Iteration: 50,000, initial Burn-in:10,000, threshold:0.001. Figure S13. Convergence of TBM-BayesCπ for SW. Iteration: 50,000, initial Burn-in:10,000, threshold:0.001. Table S1. Running time using five genomic prediction approaches in scenario 1. Table S2. Running time using five genomic prediction approaches in scenario 2. Table S3. Running time using five genomic prediction approaches in scenario 3. (ZIP 405 kb) [file 12859_2017_2003_MOESM1_ESM.zip › Supplementary file/Fig. S7 Residual variance from TBM-BayesA for ADG.png]

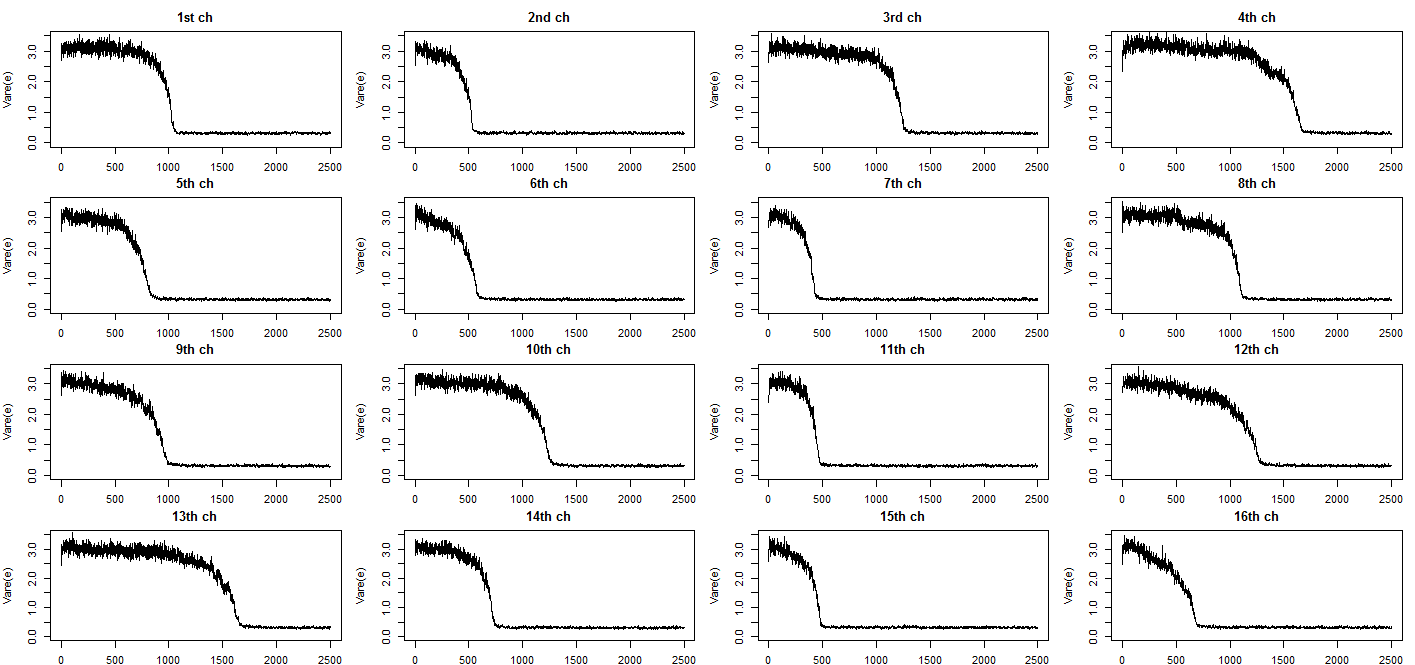

Supplement: Additional file 1: Figure S1. — Trace plots of posterior samples of residual variance from TBM-BayesA (16 chains) for SW of the first 2000 iterations. Figure S2. Trace plots of posterior samples of residual variance from TBM-BayesCπ (16 chains) for SW of the first 2000 iterations. Figure S3. Trace plots of posterior samples of residual variance from TBM-BayesA (16 chains) for CBFT of the first 1800 iterations. Figure S4. Trace plots of posterior samples of residual variance from TBM-BayesCπ (16 chains) for CBFT of the first 2500 iterations. Figure S5. Trace plots of posterior samples of residual variance from TBM-BayesA (16 chains) for CW of the first 2000 iterations. Figure S6. Trace plots of posterior samples of residual variance from TBM-BayesCπ (16 chains) for CW of the first 2000 iterations. Figure S7. Trace plots of posterior samples of residual variance from TBM-BayesA (16 chains) for ADG of the first 1400 iterations. Figure S8. Trace plots of posterior samples of residual variance from TBM-BayesCπ (16 chains) for ADG of the first 1400 iterations. Figure S9. Plot of running time in scenario1.FBM-BayesA: Fixed burn-in multiple chains parallel BayesA, TBM-BayesA: Tunable burn-in multiple chains parallel BayesA, FBM-BayesCπ: Fixed burn-in multiple chains parallel BayesCπ, TBM-BayesCπ: Tunable burn-in multiple chains parallel BayesCπ. One chain is equivalent to sequential genomic selection. Figure S10. Plot of running time in scenario 2. Figure S11. Plot of running time in scenario 3 Figure S12. Convergence of TBM-BayesA for SW. Iteration: 50,000, initial Burn-in:10,000, threshold:0.001. Figure S13. Convergence of TBM-BayesCπ for SW. Iteration: 50,000, initial Burn-in:10,000, threshold:0.001. Table S1. Running time using five genomic prediction approaches in scenario 1. Table S2. Running time using five genomic prediction approaches in scenario 2. Table S3. Running time using five genomic prediction approaches in scenario 3. (ZIP 405 kb) [file 12859_2017_2003_MOESM1_ESM.zip › Supplementary file/Fig. S8 Residual variance from TBM-BayesCa- for ADG.png]

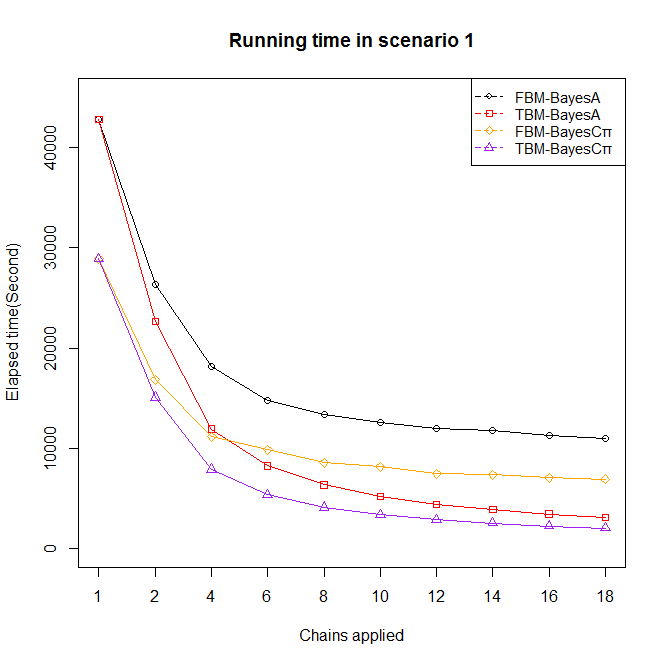

Supplement: Additional file 1: Figure S1. — Trace plots of posterior samples of residual variance from TBM-BayesA (16 chains) for SW of the first 2000 iterations. Figure S2. Trace plots of posterior samples of residual variance from TBM-BayesCπ (16 chains) for SW of the first 2000 iterations. Figure S3. Trace plots of posterior samples of residual variance from TBM-BayesA (16 chains) for CBFT of the first 1800 iterations. Figure S4. Trace plots of posterior samples of residual variance from TBM-BayesCπ (16 chains) for CBFT of the first 2500 iterations. Figure S5. Trace plots of posterior samples of residual variance from TBM-BayesA (16 chains) for CW of the first 2000 iterations. Figure S6. Trace plots of posterior samples of residual variance from TBM-BayesCπ (16 chains) for CW of the first 2000 iterations. Figure S7. Trace plots of posterior samples of residual variance from TBM-BayesA (16 chains) for ADG of the first 1400 iterations. Figure S8. Trace plots of posterior samples of residual variance from TBM-BayesCπ (16 chains) for ADG of the first 1400 iterations. Figure S9. Plot of running time in scenario1.FBM-BayesA: Fixed burn-in multiple chains parallel BayesA, TBM-BayesA: Tunable burn-in multiple chains parallel BayesA, FBM-BayesCπ: Fixed burn-in multiple chains parallel BayesCπ, TBM-BayesCπ: Tunable burn-in multiple chains parallel BayesCπ. One chain is equivalent to sequential genomic selection. Figure S10. Plot of running time in scenario 2. Figure S11. Plot of running time in scenario 3 Figure S12. Convergence of TBM-BayesA for SW. Iteration: 50,000, initial Burn-in:10,000, threshold:0.001. Figure S13. Convergence of TBM-BayesCπ for SW. Iteration: 50,000, initial Burn-in:10,000, threshold:0.001. Table S1. Running time using five genomic prediction approaches in scenario 1. Table S2. Running time using five genomic prediction approaches in scenario 2. Table S3. Running time using five genomic prediction approaches in scenario 3. (ZIP 405 kb) [file 12859_2017_2003_MOESM1_ESM.zip › Supplementary file/Fig. S9 Runtime in scenario 1.png]
